# Navigating the Complex Terrain of Obstetrics and Gynecology Malpractice: Stakeholders, Expectations, and Legal Implications

**DOI:** 10.3390/jcm14072266

**Published:** 2025-03-26

**Authors:** Lavinia Toma-Tumbar, Rodica Daniela Nagy, Marius Cristian Marinaș, Dominic Gabriel Iliescu, Monica Laura Cara

**Affiliations:** 1 Department of Ethics and Academic Integrity Legislation, Faculty of Dentistry, University of Medicine and Pharmacy of Craiova, 200349 Craiova, Romania; 2 Department of Obstetrics and Gynecology, University Emergency County Hospital Craiova, 200642 Craiova, Romania; 3 Ginecho Clinic, Medgin SRL, 200349 Craiova, Romania; monica.cara@umfcv.ro; 4 Department of Human Anatomy, Faculty of Medicine, University of Medicine and Pharmacy of Craiova, 200349 Craiova, Romania; 5 Department of Obstetrics and Gynecology, University of Medicine and Pharmacy of Craiova, 200349 Craiova, Romania; 6 Department of Public Health, University of Medicine and Pharmacy of Craiova, 200349 Craiova, Romania

**Keywords:** malpractice, litigation, obstetric malpractice, gynecology malpractice, documentation

## Abstract

This narrative review delves into the multifaceted landscape of obstetric and gynecological malpractice, focusing on stakeholders’ expectations, legal implications, and clinical considerations. Through a comprehensive analysis of the relevant literature, we evaluated 25 articles, culminating in a comprehensive understanding of the primary drivers behind malpractice litigation in this field. The review highlights the complex nature of these issues and their implications for various stakeholders. The key findings reveal the critical role of meeting medical care standards to avoid harm to patients, along with factors such as diagnostic errors, mismanagement of complications, and deficiencies in patient counseling contributing to malpractice allegations. Additionally, issues related to surgical procedures, informed consent, and documentation are explored. The review underscores the importance of collaboration, education, and accountability in mitigating the impact of malpractice and upholding patient safety in obstetric and gynecological practice.

## 1. Introduction

In obstetrics and gynecology, ensuring optimal patient outcomes remains a fundamental goal. In obstetrics, the objective of a perfect outcome, characterized by the birth of a healthy baby, stands as the paramount aspiration. However, the stark reality of malpractice in both obstetrics and gynecology often disrupts these expectations, leading to distress and disillusionment. Malpractice cases in gynecology frequently involve surgical errors, improper patient counseling, and failure to adhere to standard clinical guidelines, highlighting the need for a comprehensive evaluation of medical negligence across both fields. This article delves into the multifaceted landscape of malpractice in obstetrics and gynecology, examining the expectations placed upon healthcare professionals, the stakeholders involved, and the legal ramifications of failures to meet the standard of care [1].

Defining Malpractice in Obstetrics and Gynecology: Medical malpractice occurs when a healthcare professional, entrusted with the well-being of a patient, fails to fulfill their duty of care in a manner that leads to avoidable harm. This can manifest in various ways, including instances where a medical doctor neglects to take actions that a reasonable and prudent practitioner would undertake. For example, omitting to conduct necessary diagnostic tests or follow-up appointments could constitute medical malpractice if it results in harm to the patient. Additionally, malpractice can occur when a healthcare provider performs an action that a cautious and judicious practitioner would avoid, resulting in adverse outcomes for the patient. This could involve errors in surgical procedures, misdiagnoses, or prescribing incorrect medications. In essence, medical malpractice encompasses any deviation from the accepted standard of care that results in injury to the patient, whether through acts of commission or omission. In obstetrics, malpractice is often associated with complications arising during labor and delivery, while in gynecology, it frequently involves surgical errors and failures in patient communication regarding treatment options and risks [2,3,4,5].

To comprehensively investigate the factors contributing to indefensible claims in Obstetrics and Gynecology, this narrative review examines malpractice claims where the legal defense was unsuccessful, either due to insufficient medical documentation, clear evidence of negligence, or procedural errors in litigation, thereby shedding light on the primary causes of Obstetrics and Gynecology malpractice litigation.

## 2. Methods

The literature search covered publications from 2000 to 2024 to ensure comprehensive coverage of malpractice cases in obstetrics and gynecology. The primary databases consulted included PubMed and Scopus for medical literature, as well as the Cochrane Library, which provides systematic reviews relevant to patient safety and clinical guidelines. To integrate the legal perspective, we also reviewed Justia, a legal database containing case law and regulatory frameworks related to medical malpractice. This approach ensures a comprehensive evaluation of both medical and legal aspects of malpractice litigation in obstetrics and gynecology. We conducted a comprehensive search, employing keywords such as “malpractice”, “litigation”, “obstetric malpractice”, ‘’gynecology malpractice’’ and “documentation”, customized for the field of obstetrics and gynecology. Throughout this investigative period, we evaluated the patterns, trends, and recurring issues underlying malpractice allegations. This review integrates data from diverse sources, including clinical studies, legal cases, and legislative documents. The literature searches were conducted using medical and legal databases, focusing on obstetric malpractice, gynecological complications, and medicolegal aspects. Key themes were identified, including stakeholder perspectives, malpractice definitions, and legal frameworks. The aim was to provide a thorough examination of the circumstances surrounding these claims, facilitating a deeper understanding of the challenges and shortcomings within Obstetrics and Gynecology practice and healthcare delivery. The study received approval from the Institutional Ethics Committees before initiation, approval No. 31/22.03.2021.

## 3. Results

During our study, an evaluation of 84 articles was conducted to discern the primary causes of Obstetrics and Gynecology malpractice litigation in OB–GYN departments. Out of the 84 articles reviewed, 25 pertinent to the subject matter were selected and referenced in this article. Analysis of the literature revealed several significant findings.

### 3.1. Obstetrics

The review of the literature on obstetric malpractice highlights several critical factors contributing to malpractice (presumed or real) litigation, revealing a complex interplay of medical, legal, and ethical concerns.

*Failure to Meet Medical Standards*: A predominant cause of obstetric malpractice claims is the failure of obstetricians to adhere to established medical care standards. These standards serve as benchmarks for safe and effective care, ensuring that patients receive optimal treatment based on current evidence. Non-compliance with clinical guidelines can result in preventable harm to both the mother and the child, leading to adverse outcomes, including maternal mortality and neonatal injuries. Research has identified systemic factors contributing to deviations from these standards, including organizational inefficiencies, inadequate resources, and gaps in medical training. The relevance of this review is to emphasize the contributing factors underlying such non-compliance, rather than merely listing malpractice claims [6]. Despite the availability of well-established clinical guidelines aimed at standardizing obstetric care, non-adherence remains prevalent. This deviation from recommended practices has been consistently associated with negative patient outcomes and subsequent malpractice claims [6].

*Diagnostic-Related Errors*: These represent a substantial proportion of malpractice claims in obstetrics. Diagnostic errors, defined as delayed, missed, or incorrect diagnoses by obstetricians or gynecologists, can significantly impact patient outcomes. Conditions such as ectopic pregnancy, preeclampsia, and fetal distress require timely and accurate diagnosis to prevent severe complications. Misdiagnosis or delayed recognition of fetal distress, for instance, can result in hypoxic–ischemic encephalopathy, leading to lifelong disabilities or neonatal death [7]. The literature suggests that up to 70% of obstetric claims stem from such complications. Additionally, perinatal diagnosis of fetal anomalies has been implicated in malpractice litigation, often associated with errors in obstetric ultrasound [8]. The role of medical equipment and devices in mitigating these errors must be further explored. This discussion should also address how the organization and administrative roles within healthcare facilities influence diagnostic accuracy and patient outcomes.

*Mismanagement of Complications During Labor:* Errors during labor and delivery remain a substantial source of malpractice claims, with consequences ranging from mild complications to catastrophic outcomes. Specific complications such as shoulder dystocia and delayed intervention during fetal distress are frequently cited in litigation. Inaccurate interpretation of fetal monitoring (cardiotocography), improper maneuvering in cases of dystocia, and failure to timely initiate a cesarean section, when indicated, contribute to legal disputes [9]. Studies indicate that poor outcomes, such as cerebral palsy, are often linked to failures in managing these complications, making them central to malpractice allegations [10,11]. Adherence to active labor management protocols and continuous fetal monitoring are essential preventive measures. Domingues et al. (2015) reviewed adverse events in labor management and found that among the frequently reported obstetrical situations leading to prosecution in medico-legal cases were perinatal asphyxia (50% cases), traumatic lesions in newborn-like instrumented deliveries, shoulder dystocia, vaginal delivery in breech presentation (24%), and maternal sequelae (19%) [8].

*Inadequate Patient Counseling and Informed Consent*: Effective communication between obstetricians and patients is critical in minimizing malpractice claims. Deficiencies in counseling, particularly regarding alternative delivery methods (e.g., vaginal birth versus cesarean section), contribute to patient dissatisfaction and legal disputes. Patients frequently report feeling inadequately informed about procedural risks, leading to allegations of medical negligence. Studies indicate that shared decision-making significantly reduces litigation risk [12]. The American College of Obstetricians and Gynecologists (ACOG) guidelines stress that informed consent should be a dynamic and transparent process rather than a mere legal formality [11]. Studdert et al. (2006) reported that in 30% of gynecological malpractice cases, plaintiffs claimed they were not given adequate information about risks associated with procedures such as hysterectomies or sterilization procedures [13].

*Improper Documentation*: Inadequate or incomplete documentation remains a major concern in obstetric malpractice. Proper documentation of medical events, interventions, and decision-making processes is crucial for legal defense. A study showed that 54% of shoulder dystocia claims were ruled against the physician due to poor or incomplete documentation, underscoring the importance of accurate record-keeping in reducing litigation [14].

Several systemic factors contributing to these failures have been identified, including delays in care, inadequate training, poor interprofessional communication, and resource constraints [15]. Ghaith et al. (2022) reported that trainees in obstetrics and gynecology often lacked the experience to handle complex obstetric emergencies, resulting in deviations from standards [16].

### 3.2. Gynecology

Gynecological malpractice often centers on surgical procedures and patient autonomy. The review of malpractice cases in gynecology reveals distinct patterns reflecting the unique risks associated with gynecological care.

*Failed Sterilization Procedures*: A commonly cited issue in gynecological malpractice is unsuccessful sterilization, representing 19% of all gynecological lawsuits in the United States [17]. Such cases often involve allegations of negligence in surgical technique, leading to unintended pregnancies. These outcomes not only impose psychological and financial burdens on patients but also present significant medico-legal challenges. The impact of the outcome of these procedures, as opposed to the general duty of care in the context of assistance, should also be discussed in the discussion section. This distinction is crucial, as it almost seems like a result of obligation rather than a duty of care.

*Surgical Errors Leading to Organ Damage*: Gynecological surgeries carry inherent risks, including injury to the urinary tract, bowel, bladder, pelvic vessels, and nerves. The consequences of surgical errors extend beyond medical complications to include prolonged hospital stays, additional surgeries, emotional distress, and financial burdens [18].

*Retained Surgical Instruments*: Identified as a recurrent surgical error leading to litigation, retained surgical instruments underscore the importance of clinical risk management. The impact of clinical risk management activities and the reduction in sentinel/system events over time should also be discussed in the discussion section, as these factors play a crucial role in mitigating malpractice claims and improving patient safety.

*Improper Documentation*: Similar to obstetrics, inadequate documentation in gynecological cases contributes to malpractice litigation. Studies have shown that 24% of claims were deemed indefensible due to incomplete records [19], particularly in cases involving sterilization, uterine injuries, or post-operative complications [20]. Ensuring detailed and accurate documentation can prevent a substantial number of lawsuits [20]. Additionally, the role of competence monitoring and continuous training in litigation prevention should be emphasized. Regular assessment of healthcare professionals’ documentation skills, alongside targeted training programs, may significantly reduce malpractice claims related to incomplete or inaccurate records. Awareness of published guidelines might not be enough to improve the quality of documentation [20].

*Failure to Adhere to Clinical Guidelines*: A study by Ravlo et al. evaluated approved claims for compensation in gynecological patients in Norway and emphasized that guidelines or good clinical practice were not followed in 40% of cases [21].

### 3.3. Contraception and IUD Complications

The use of intrauterine devices (IUDs) and hormonal contraception has been associated with medicolegal disputes, particularly when complications arise due to improper insertion techniques, failure to recognize adverse effects, or inadequate patient counseling. Among these, uterine perforation is a significant issue, with legal implications when linked to procedural negligence. Grimaldi et al. (2005) documented an unusual uterine perforation involving the Multiload-Cu 375R, emphasizing the forensic and legal considerations of such cases [22]. Additionally, failure to adequately assess contraindications for hormonal contraception, particularly in patients with a history of thromboembolism, cardiovascular diseases, or hormone-sensitive cancers, has led to litigation. Cases involving deep vein thrombosis (DVT) following contraceptive administration have been reported, often linked to insufficient patient screening and counseling. The risk of legal claims increases when patients are not thoroughly informed about potential complications or when informed consent documentation is incomplete. Ensuring adherence to standardized guidelines, improving documentation practices, and enhancing patient-centered counseling are essential strategies for reducing malpractice claims related to contraception and IUD complications [22].

The analysis of 500 medico-legal cases (225 obstetric and 275 gynecological) revealed several key causes of disputes. Misguided allegations were the most prevalent, accounting for 46% of cases; these claims frequently arose due to inappropriate advice given to the plaintiff, encouraging them to seek legal counsel. Incompetent management contributed to 19% of cases, involving inadequate clinical decision-making, poor teamwork, and professionals working beyond their expertise. Errors in judgment were present in 12% of cases, frequently associated with inappropriate alternative management strategies, failure to escalate cases to senior colleagues, or lack of specialist input during complex procedures. Lack of expertise was a factor in 9% of disputes, highlighting cases where practitioners held the necessary qualifications but lacked technical competence or failed to seek assistance when needed. Failure of communication was identified in 7% of cases, including poor counseling, lack of informed consent, discrepancies between verbal and written information provided to patients, and failure to warn of procedural risks. Poor supervision contributed to 6% of disputes, involving inadequate oversight, monitoring, or guidance of medical staff, leading to adverse outcomes. Inadequate staffing was the least common cause, identified in only 1% of cases, likely reflecting situations where limited personnel or resources compromised patient care and safety, leading to disputes [10].

Malpractice and medical negligence remain critical concerns, particularly in obstetrics, where up to 70% of claims are linked to abnormalities in cardiotocography. These cases encompass situations where healthcare professionals fail to adhere to the standard of care, resulting in preventable injuries. The most common obstetric cause of disputes in this analysis was intrapartum hypoxic–ischemic encephalopathy [10]. This condition frequently resulted from inadequate interpretation of labor warning signs, delayed identification of fetal distress, insufficient anticipation and handling of shoulder dystocia, and improper application of maneuvers during instrumental delivery. In cerebral palsy-related cases, notable causes included intrapartum hypoxic encephalopathy and various forms of cerebral injury. Misguided allegations were present in 53% of obstetric cases related to cerebral palsy, indicating that a significant proportion of claims lacked valid medical grounds. Neonatal death was another significant factor, accounting for 10% of obstetric disputes, raising concerns about the quality of perinatal care provided during labor and delivery. Developmental delay was implicated in 6% of obstetric cases, often associated with allegations of negligence or substandard care during pregnancy or childbirth, potentially leading to long-term consequences for the newborn [10]. Improper patient counseling was another recurring issue, responsible for 7% of disputes. Inadequate counseling often resulted in unrealistic patient expectations, misunderstandings, and dissatisfaction, ultimately leading to malpractice complaints [10]. Effective patient counseling requires not only the accurate presentation of diagnosis, treatment options, risks, and benefits but also ensuring that patients fully comprehend the provided information. Communication failures in this area may have contributed to allegations of negligence, particularly in cases where instrumental deliveries were performed despite a potentially safer option, such as cesarean section [10] (Table 1).

## 4. Discussions

For every expectant parent, the anticipation of welcoming a perfect baby into the world is palpable. This aspiration extends beyond mere physical health to encompass the entirety of the birthing experience. Given the high expectations placed on obstetricians and gynecologists, ensuring patient safety through structured risk management becomes paramount in mitigating litigation risks. Any deviation from this ideal scenario not only leads to distress and disappointment but may also catalyze litigation, particularly when negligence is perceived or suspected. Beyond pregnancy and childbirth, gynecological care plays a crucial role in women’s health, addressing conditions that, if mismanaged, can lead to serious medical, legal, and ethical implications [1].

A multidisciplinary approach involving healthcare providers, risk management teams, and legal professionals is crucial in minimizing malpractice exposure. Each group brings its own perspectives, interests, and motivations to the forefront of the medical–legal debate. Patients, alongside their families and the media, often serve as vocal advocates for accountability and transparency in both obstetric and gynecologic care. Healthcare providers, including obstetricians, gynecologists, nurses, and paramedical staff, must navigate the burden of meeting clinical standards while managing complex medical cases ranging from high-risk pregnancies to gynecological surgeries and reproductive health interventions. Healthcare facilities play a central role in this ecosystem, as they provide the infrastructure, equipment, and organizational framework necessary for safe medical practice. The implementation of general clinical risk management strategies within these institutions is critical in mitigating malpractice risks. Such strategies include standardized protocols for obstetric and gynecologic emergencies, continuous staff training, auditing of adverse events, and fostering a culture of patient safety. Risk management departments within hospitals work closely with medical personnel to monitor compliance with best practices, optimize resource allocation, and ensure that both technological and human factors contribute to high-quality care delivery. Effective clinical governance structures within healthcare organizations are essential in reducing errors and improving patient outcomes [1].

The Role of Organizational, Process, and Equipment Factors in Malpractice Risks.

While malpractice discussions often focus on individual competence, an equally important aspect involves organizational structures, healthcare processes, and the role of medical devices and equipment in patient safety. Several studies have highlighted systemic failures as a significant factor in medical errors, including suboptimal hospital protocols, lack of access to necessary equipment, and deficiencies in staff coordination [6,15].

### 4.1. Equipment and Device-Related Issues

Malpractice claims in OB–GYN frequently involve misuse, malfunction, or unavailability of critical medical devices such as cardiotocography monitors, vacuum extractors, and laparoscopic instruments. For example, cardiotocography interpretation errors contribute to up to 70% of obstetric malpractice cases [7]. Additionally, failures in surgical equipment, including defective suturing devices or instrument misalignment in laparoscopic procedures, have been implicated in adverse outcomes leading to legal disputes [18]. Regular maintenance, staff training, and adherence to equipment safety protocols are essential in mitigating these risks.

### 4.2. Process Failures in OB–GYN Malpractice

Beyond individual actions, breakdowns in clinical processes such as delayed emergency response times, inadequate staffing, and inefficient triage systems contribute to patient harm. Studies have demonstrated that prolonged decision-to-incision times in emergency cesarean sections increase both neonatal morbidity and the likelihood of litigation [11]. In gynecological surgeries, failure to follow standardized surgical protocols has been associated with higher rates of intraoperative injuries and malpractice claims [19]. Implementing robust hospital-wide clinical governance frameworks, including real-time performance auditing and structured communication tools, is crucial in addressing these issues.

### 4.3. Organizational Culture and Safety Strategies

A safety-driven organizational culture is integral to reducing malpractice risks. Institutions that prioritize interdisciplinary collaboration, open error reporting, and continuous quality improvement have been shown to reduce adverse events and litigation rates [23].

Electronic Health Records (EHRs) are widely recognized for improving documentation accuracy, reducing errors, and enhancing legal defensibility in malpractice cases. However, their effectiveness is highly dependent on how they are integrated into clinical workflows, the training of healthcare professionals, and the continuous adaptation of digital tools to real-world medical practice.

Despite their benefits, EHR implementation alone does not eliminate medico-legal risks. Studies have shown that omissions, distractions, and workarounds occur frequently when EHRs are not optimally designed or adapted to clinical needs [24]. For instance, auto-fill functions, template use, and alert fatigue can contribute to errors when clinicians rely on pre-populated information without critical review. Additionally, real-time adaptation of medical guidelines to individual patient cases remains a clinician-dependent process, meaning that EHRs must be paired with ongoing staff training to prevent misinterpretations and documentation failures. From an organizational perspective, successful EHR integration requires:

- Comprehensive user training to ensure physicians, nurses, and administrative staff are proficient in using the system efficiently;
- Regular process revisions and updates to ensure that clinical guidelines and decision-support tools remain relevant;
- Customizable EHR interfaces minimize distractions and cognitive overload, allowing clinicians to focus on patient-centered care rather than excessive data entry;
- Risk management audits to assess whether documentation standards are being met and to identify patterns of omission or misrepresentation that could increase legal exposure.

Furthermore, healthcare institutions must acknowledge that EHR use is not just a technological solution but a component of a broader patient safety culture. Regular audits, revision of workflows, and continuous professional development are essential to ensure that digital documentation enhances rather than complicates medical practice [24].

Given the complexity of malpractice in OB–GYN, future risk-reduction strategies should emphasize not only clinician competence but also systemic improvements in hospital management, process optimization, and equipment reliability to enhance overall patient safety and minimize litigation exposure.

Insurance companies play a pivotal role in assessing risk and providing financial coverage, while legal practitioners serve as arbiters of justice, representing both plaintiffs and defendants in malpractice cases. Risk management strategies must move beyond compliance and focus on proactive interventions, such as real-time auditing and structured decision-making protocols [1].

Malpractice litigation not only affects individual practitioners but also contributes to defensive medicine and rising healthcare costs. Plaintiffs seek redress for their suffering, whether related to birth-related injuries, surgical complications, or misdiagnosed gynecological conditions, while defendants grapple with the threat to their professional reputation and financial stability. Insurance companies must balance protecting their interests with fulfilling their obligations to policyholders. Legal practitioners, armed with expertise in medical malpractice law, advocate for their clients’ rights while striving for equitable resolutions. The intersection of obstetric and gynecologic care with medical malpractice highlights the complexity of maintaining high standards while addressing legal and ethical concerns in women’s health [1].

The repercussions of obstetric malpractice extend far beyond the confines of the delivery room, often culminating in protracted legal battles with profound implications for all parties involved. Plaintiffs seek redress for their suffering, while defendants grapple with the threat to their professional reputation and financial stability. Insurance companies navigate the delicate balance between protecting their interests and upholding their obligations to policyholders. Legal practitioners, armed with expertise in medical malpractice law, advocate for their clients’ rights while striving for equitable resolutions.

Understanding malpractice trends requires analyzing both preventable errors and systemic failures within healthcare institutions. This can manifest in various ways, including instances where a medical doctor neglects to take actions that a reasonable and prudent practitioner would undertake. For example, omitting to conduct necessary diagnostic tests or follow-up appointments could constitute medical malpractice if it results in harm to the patient. Additionally, malpractice can occur when a healthcare provider performs an action that a cautious and judicious practitioner would avoid, resulting in adverse outcomes for the patient. This could involve errors in surgical procedures, misdiagnoses, or prescribing incorrect medications. In essence, medical malpractice encompasses any deviation from the accepted standard of care that results in injury to the patient, whether through acts of commission or omission. According to the information provided in the one article, the primary contributors to maternal mortality include thromboembolism and pregnancy-induced hypertension, with subsequent significant factors being early pregnancy loss, hemorrhage, amniotic fluid embolism (AFE), and genital tract sepsis [25].

The findings underscore several systemic challenges in obstetric and gynecologic care, particularly in communication, clinical expertise, and risk assessment. While some disputes were grounded in genuine medical errors, the high percentage of misguided allegations suggests that a substantial number of claims may have been driven by misinformation or misinterpretation of clinical outcomes [10]. This highlights the need for enhanced medico-legal education for both healthcare providers and patients to mitigate unnecessary litigation.

Additionally, the limitations of retrospective medico-legal analyses must be acknowledged. Variations in national legal frameworks, subjective interpretations of medical negligence, and differences in clinical practice standards may influence case outcomes. Furthermore, while this study identifies prevalent themes in malpractice claims, it does not assess whether these claims resulted in specific policy changes or improvements in clinical practice. Future research should focus on integrating comprehensive clinical risk management strategies within healthcare organizations to address the root causes of disputes, improve patient safety, and minimize litigation risks [10].

Healthcare providers may opt for instrumental delivery without due consideration of the risks involved, even though cesarean section (CS) may have been a more suitable delivery method in certain situations. Improper patient counseling emerged as a contributor, accounting for 7% [10]. Inadequate or improper counseling can lead to misunderstandings, unrealistic expectations, and dissatisfaction among patients, potentially culminating in malpractice complaints. Effective patient counseling involves not only conveying information about diagnosis, treatment options, risks, and benefits but also ensuring that patients have a thorough understanding of their condition and the proposed course of action. It requires clear and transparent communication tailored to the individual patient’s needs, preferences, and cultural background.

One study aimed to investigate whether obstetricians’ choices regarding delivery methods are influenced by their risk tolerance and perceptions of litigation and malpractice risks. Conducted through a nationwide survey of Norwegian obstetricians, the study utilized clinical scenarios to assess the choice of delivery method. The results showed considerable variation in obstetricians’ willingness to consent to cesarean requests across scenarios. Perceived risk of complaints and litigation emerged as a significant factor influencing obstetricians’ decisions in favor of cesarean delivery, while no association was found with risk attitude. Overall, this suggests that obstetricians’ judgments regarding cesarean requests in ambiguous clinical cases are notably influenced by concerns about legal repercussions and complaints [26].

Addressing the root causes of malpractice and medical negligence requires a multifaceted approach, encompassing ongoing education, adherence to clinical guidelines, and a culture of accountability and transparency within healthcare institutions. By prioritizing patient-centered care and fostering a collaborative environment that values continuous improvement, obstetric providers can strive to minimize the occurrence of adverse events and uphold the trust placed in them by expectant mothers and their families [26].

Legal disputes related to failed sterilization procedures emphasize the critical role of proper patient counseling, informed consent, and adherence to surgical protocols to minimize medico-legal risks. These cases likely involved allegations of unsuccessful sterilization procedures, raising concerns about the effectiveness of the procedure and its impact on the patient’s reproductive health. Cases involving perforated uterus accounted for 8% of gynecological disputes. These cases may have included allegations of surgical errors or complications during procedures involving the uterus, leading to adverse outcomes and disputes. Urinary tract injuries were identified in gynecological cases, indicating complications related to gynecological surgeries or procedures. These cases may have raised concerns about the quality of surgical care and potential long-term consequences for the patient’s health. Endoscopic surgery injuries lead to disputes. Misguided allegations contribute significantly. Instances of uterine or organ perforation during surgeries, as well as injuries to the urinary tract or major blood vessels, underscored the importance of precise surgical technique and meticulous attention to detail in minimizing adverse outcomes [10].

Among the contentious issues in gynecological practice are hysterectomy with doubtful indications and the wrongful removal of ovaries during hysterectomy without appropriate consent. These practices raise ethical and legal concerns, as they may result in irreversible consequences for patients, including infertility, hormonal imbalances, and psychological distress.

Hysterectomy, a major surgical procedure involving the removal of the uterus, should only be performed when medically necessary and after thorough consideration of alternative treatment options. However, cases of hysterectomy with doubtful indications suggest potential overutilization or inappropriate recommendations by healthcare providers. Such practices not only pose risks to patients’ physical health but also raise questions about the adequacy of informed consent and patient autonomy.

Furthermore, the wrongful removal of ovaries during hysterectomy without appropriate consent represents a breach of patient trust and autonomy. Ovaries play a crucial role in hormone production and reproductive health, and their removal can have profound implications for a patient’s well-being. When there is uncertainty about whether ovarian removal is necessary, healthcare providers must engage in transparent discussions with patients, ensuring that they fully understand the risks and benefits of the procedure before consenting [27].

To address these issues, gynecologists must adhere to established clinical guidelines and ethical principles, prioritizing patient-centered care and informed decision-making. This requires open communication, comprehensive preoperative counseling, and facilitating collaborative decision-making processes that let patients actively engage in their care and treatment. Additionally, healthcare institutions should implement robust systems for quality assurance, peer review, and continuous education to minimize the occurrence of disputed events and enhance the overall quality and safety of gynecological practice.

Poor documentation is a pervasive issue in both obstetrics and gynecology, compromising legal defense and leading to unfavorable judgments in malpractice cases. Thorough documentation of events is essential to avoid such litigation [28].

Enhancing documentation practices, including structured templates and electronic health records, can reduce the risk of indefensible malpractice claims [19]. Incomplete and inaccurate documentation of relevant events compromised the legal defense and influenced unfavorable legal decisions, underscoring the importance of comprehensive and precise record-keeping practices in Obstetrics. Inadequate documentation significantly impacts the outcome of malpractice claims. For instance, in cases of shoulder dystocia, where prompt and appropriate management is crucial, poor documentation of the steps taken to address the condition often results in judgments against physicians. In fact, another study found that shoulder dystocia claims were decided against the physician because of poor documentation [29].

Similarly, cases involving uterine rupture are deemed indefensible when signs are misinterpreted or when a physician is not immediately available to intervene. These cases highlight the critical importance of timely and accurate documentation in obstetric emergencies [30,31].

Complications such as injury to the ureter or bladder, though not frequent, carry a high rate of litigation. In a Canadian report on urinary tract injuries during benign gynecologic surgery, the majority of the cases resulted in litigation [18]. Some cases were considered indefensible due to the physician’s failure to recognize and manage these injuries in a timely and appropriate manner [30].

Unfortunately, residency training frequently overlooks adequate medicolegal education, heightening trainee apprehension. While trainees gain valuable experience from participating in high-risk procedures and acquiring necessary skills, programs must ensure sufficient supervision and documentation to verify the trainees’ proficiency levels. This underscores the need for comprehensive medicolegal education within residency programs to equip trainees with the necessary skills to navigate complex clinical scenarios while prioritizing patient safety and legal compliance [32].

Responsibility for malpractice can be of a penal or civil nature. Even in a criminal trial, the liability of the physician and/or the healthcare unit will be a pecuniary liability in the form of financial compensation to cover the moral and material damages suffered by the victims [33].

Most often, in criminal matters, in addition to the custodial sentence, the courts impose on the accused, the physician, the security measure consisting of the prohibition of the right to practice medicine in the obstetrics-gynecology specialty. The purpose of this measure is to eliminate the potential danger that the accused would represent in the exercise of the medical profession [34].

Medical malpractice liability is subject to both civil and administrative law in many jurisdictions. In several European legal systems, including Romania, France, and Germany, when a physician is employed by a public or private healthcare institution, both the individual practitioner and the institution may be held jointly liable for malpractice claims. This means that hospitals or clinics may be required to compensate victims alongside the physician if the malpractice occurred within the scope of employment.

Under Romanian malpractice law, for instance, Law No. 95/2006 on healthcare reform establishes that both medical professionals and healthcare providers (hospitals, clinics, or private practices) share liability for damages in malpractice cases. The medical unit’s liability may arise due to organizational failures, lack of proper equipment, insufficient staffing, or failure to implement patient safety protocols.

This legal principle is consistent with jurisprudence in other EU countries, where medical units bear responsibility not only for their employees’ professional conduct but also for ensuring institutional compliance with medical safety standards. Therefore, in civil malpractice claims, compensation for damages can be pursued against both the physician and the healthcare institution, ensuring that patients receive financial redress for harm suffered due to medical negligence [35].

In that context, it is important to outline the key strategies for reducing malpractice risks in OB–GYN practice. In our view, the most relevant strategies should be addressed to enhance clinical competence, improve communication, strengthen documentation practices, promote a culture of patient safety, and implement legal and risk management strategies.

The failure to meet medical standards in OB–GYN is multifactorial, encompassing individual, systemic, and contextual elements. While diagnostic errors, inadequate management of labor, and guideline non-adherence are common contributors, addressing these issues requires a combination of education, system redesign, and robust safety protocols. Future research should focus on identifying barriers to guideline implementation and developing interventions tailored to high-risk settings. Clinical competence could be improved by

- Simulation-based training—Regular simulation training for obstetric emergencies, such as shoulder dystocia, postpartum hemorrhage, and eclampsia, enhances clinicians’ readiness to manage critical scenarios, improves team performance during obstetric emergencies, and reduces errors [5,36,37];
- Using checklists and protocols: surgical safety checklists decreased complications and enhanced compliance with medical standards [37,38];
- Adherence to Evidence-Based Guidelines—Strict adherence to clinical guidelines, such as those published by the ACOG, ensures standardized high-quality care [3,11];
- Enhanced Documentation: accurate documentation contributes to defending malpractice claims and improving patient care quality [13];
- Interprofessional/Interdisciplinary communication: structured communication tools and reduced miscommunication-related errors [23];
- Continuous education and credentialing for medical personnel—requiring OB–GYN practitioners to participate in regular continuing medical education (CME) and periodic re-credentialing ensures that providers stay current with evolving medical standards and practices.

Inadequate patient counseling and informed consent in OB–GYN remain significant challenges despite well-established guidelines. The reviewed literature highlights the multifactorial nature of this issue, encompassing provider communication skills, systemic barriers, and patient-related factors. Addressing these challenges requires a combination of provider education, patient-centered tools, and institutional policies aimed at fostering shared decision-making. Strategies for improvement in communication in obtaining informed consent involves enhanced communication skills training that improves provider confidence and effectiveness in delivering complex information. (e.g., Makoul and Clayman (2006) recommend using the SPIKES model (Setting, Perception, Invitation, Knowledge, Emotions, Strategy) to guide difficult conversations) [12,39]. The use of decision aids like visual aids, pamphlets, and digital tools can help patients better understand their options. A study by Stacey et al. (2014) demonstrated that decision aids improved patient knowledge and reduced decisional conflict in OB–GYN settings [40]. Also, the implementation of checklists for informed consent ensures that all essential topics are covered. Haynes et al. (2009) reported that surgical safety checklists reduced errors and improved documentation [38]. The importance of cultural competence training for healthcare teams was identified as a standard of care (Betancourt et al. (2005)) [41]. Another relevant issue refers to providing interpreters and culturally tailored counseling that can facilitate understanding among diverse patient populations.

Comprehensive documentation of the counseling and consent process provides legal protection for providers and clarity for patients [42].

Strengthening documentation practices must imply using comprehensive and timely medical records that should reflect clinical reasoning, informed consent discussions, and follow-up plans as well as the use of Electronic Health Records (EHR) with built-in alerts and prompts that can improve adherence to guidelines and reduce omissions [24].

Strategies to mitigate litigation risks also refer to the implementation of a proactive risk management system aiming to identify and address potential safety issues before they result in harm and an accurate and thorough documentation that provides critical evidence in defense against malpractice claims [13,43]. Early disclosure of adverse events and sincere apologies can reduce the likelihood of litigation; many patients pursue lawsuits due to dissatisfaction with communication rather than the event itself [44]. Lastly, defensive medicine mitigation must be addressed because avoiding unnecessary interventions driven by fear of litigation reduces complications and aligns care with evidence-based practices. Defensive medicine practices often paradoxically increase malpractice risk [45].

### 4.4. Comparison with Other Surgical Settings

While obstetric and gynecologic malpractice claims have distinct characteristics, they share common themes with other high-risk surgical fields such as general surgery, orthopedic surgery, and neurosurgery. Across these specialties, common causes of litigation include surgical complications, errors in clinical judgment, lack of informed consent, and documentation failures [13]. Studies have shown that technical errors, particularly in minimally invasive and laparoscopic procedures, are significant contributors to malpractice claims, both in OB–GYN and other surgical fields [18].

For instance, failed sterilization and uterine perforation are common medico-legal issues in gynecology, paralleling disputes in general surgery involving unintended bowel or vascular injuries during laparoscopic interventions [19]. Similarly, urinary tract injury cases are frequently cited in litigation related to colorectal and urological surgeries, emphasizing the need for heightened intraoperative vigilance and postoperative monitoring to prevent long-term morbidity [18].

Poor documentation is another recurrent factor in malpractice claims across surgical disciplines. In OB–GYN, inadequate documentation was found especially in gynecological cases, often leading to indefensible claims [19]. Similarly, in orthopedic and general surgery, incomplete operative notes and failure to document informed consent have been cited as major contributors to unfavorable legal outcomes [29]. A study analyzing surgical malpractice litigation highlighted that cases with insufficient documentation were significantly more likely to result in judgments against the physician, underscoring the necessity for meticulous record-keeping across all surgical disciplines [13].

Risk mitigation strategies from other high-risk specialties, such as cardiothoracic surgery and neurosurgery, may offer valuable insights for OB–GYN practice. For example, the widespread adoption of standardized surgical safety checklists in general surgery has led to reductions in surgical complications and malpractice claims, suggesting that similar structured approaches could enhance patient safety in obstetrics and gynecology [37,38]. Additionally, simulation-based training, which has demonstrated effectiveness in reducing surgical errors in trauma and emergency surgery, could be further integrated into OB–GYN training programs to improve preparedness for obstetric emergencies and high-risk gynecological procedures [36].

Given these parallels, further research should explore the applicability of evidence-based risk reduction strategies from other surgical specialties to OB–GYN practice, with the aim of improving patient outcomes and minimizing litigation risks.

Competence Monitoring and Training in Litigation Prevention

Ensuring that healthcare professionals undergo continuous training and competence monitoring is crucial in mitigating malpractice risks. Regular assessment of documentation skills, adherence to clinical guidelines, and hands-on simulation-based training can significantly improve decision-making and reduce errors leading to litigation. Healthcare institutions should establish standardized training programs that integrate real-case malpractice scenarios to help providers recognize and prevent potential legal issues. Competence evaluation should also be linked to credentialing and re-credentialing processes to reinforce adherence to best practices.

The Role of Equipment and Devices in Mitigating Diagnostic Errors

Medical equipment and diagnostic tools play a pivotal role in reducing errors and ensuring accurate diagnoses in obstetrics and gynecology. Advanced fetal monitoring systems, improved ultrasound imaging, and AI-driven decision support tools help enhance the early detection of complications such as fetal distress or ectopic pregnancies. However, the effectiveness of such devices is directly linked to the organization and administration within healthcare facilities. Standardized protocols, proper equipment maintenance, and staff training on technological advancements are necessary to optimize patient outcomes and minimize litigation risks. Institutions should also implement protocols that ensure medical staff are proficient in using these technologies, as improper handling or misinterpretation of data can contribute to diagnostic errors.

The Impact of Failed Sterilization Outcomes vs. the General Duty of Care

Failed sterilization procedures raise ethical, legal, and financial concerns for patients and providers alike. Unlike other medical procedures where outcomes may vary, patients undergoing sterilization often perceive the procedure as a guaranteed solution to avoid future pregnancies. This has led to litigation cases where the failure of sterilization is argued as a breach of duty of care, bordering on result obligation rather than a standard healthcare duty. Courts have differed in interpreting liability in such cases, depending on the jurisdiction and medical evidence presented. In the United States, sterilization failure represents 19% of gynecological malpractice lawsuits [17]. A comparative analysis of different legal systems could provide insights into how various regions address failed sterilization claims and the compensation frameworks in place.

Clinical Risk Management Activities and Sentinel/System-Event Reduction Over Time

Healthcare institutions must implement robust clinical risk management systems to prevent sentinel events and reduce malpractice claims [29]. Sentinel events such as retained surgical instruments, mismanagement of labor complications, or failure to act on fetal distress signals can be mitigated through institutional policies, checklists, and real-time monitoring protocols. Data from clinical risk management programs indicate that facilities employing structured risk assessments and proactive interventions experience a significant decline in preventable errors. Implementing system-wide changes, such as mandatory reporting of near-miss events and team-based communication strategies, further strengthens patient safety and legal defense mechanisms.

Legal Frameworks, Alternative Dispute Resolution, and Systemic Issues

The litigation process in obstetric and gynecologic malpractice cases varies significantly depending on jurisdiction, legal frameworks, and the approach to medical liability. In common law systems such as the United States and the United Kingdom, malpractice cases often follow an adversarial legal process, requiring extensive discovery phases, expert witness testimonies, and, in many cases, jury trials [13,46]. In contrast, civil law countries such as France, Germany, and Italy typically employ judge-led investigations and administrative compensation mechanisms, which can result in more predictable case outcomes [23,35].

Alternative dispute resolution (ADR) mechanisms, including mediation and arbitration, have gained prominence as strategies to resolve malpractice claims outside traditional court systems. Studies suggest that ADR can reduce litigation costs, expedite case resolutions, and enhance patient satisfaction with legal outcomes [19,21]. However, its effectiveness is highly dependent on national legal cultures—some healthcare systems, particularly in Northern Europe, favor negotiated settlements, whereas others, like the United States, maintain a strong preference for courtroom proceedings [24].

Additionally, distinctions between civil and criminal liability significantly influence malpractice litigation. While most cases result in civil lawsuits that lead to financial compensation for damages, egregious cases of negligence may escalate to criminal charges. In Italy and Romania, for instance, there has been a rising trend of criminalizing medical errors, increasing the legal risks for healthcare professionals [29,35]. This evolution raises concerns about striking a balance between patient rights and ensuring fair legal protections for physicians. As medical malpractice laws continue to evolve, legal reforms should aim to standardize dispute resolution mechanisms, promote alternative legal solutions, and create safeguards to protect both patients and healthcare providers from excessive legal exposure [13,23].

Barriers and Limitations in Malpractice Risk Reduction

Despite significant advancements in clinical safety protocols, medico-legal education, and risk management strategies, reducing malpractice claims in obstetrics and gynecology remains challenging. A review of the literature identifies several barriers and limitations that affect the effectiveness of malpractice prevention efforts.

The legal and policy landscape surrounding malpractice claims is marked by significant variability across jurisdictions, resulting in inconsistencies in case rulings and compensation structures [35]. This divergence complicates the legal process and contributes to defensive medicine practices, where the fear of litigation leads physicians to perform unnecessary interventions, such as elective cesarean sections without clear medical indications [34]. Furthermore, proving negligence in obstetric malpractice cases remains a complex and time-consuming process, requiring extensive medical–legal evaluations, expert testimonies, and prolonged legal proceedings, often delaying both patient compensation and professional accountability [13]. Beyond legal considerations, organizational and systemic barriers also influence malpractice risks. While standardized clinical protocols exist—such as those for fetal monitoring and emergency cesarean sections—their implementation remains inconsistent, leading to variations in patient outcomes [3,11].

The lack of access to structured simulation-based training further compounds the issue, as many hospitals do not provide routine emergency preparedness programs, which are critical for managing obstetric emergencies [36]. Additionally, understaffing and heavy workloads increase communication failures and delays in clinical decision-making, further elevating medico-legal risks [19].

From a technological and documentation perspective, Electronic Health Records (EHRs) offer improvements in medical documentation, yet usability challenges persist, including alert fatigue, incomplete data entry, and workflow disruptions that can compromise patient safety [24].

Another key challenge arises from patient expectations and communication barriers. Increased awareness of medical litigation has heightened patient expectations, sometimes leading to legal claims even when no demonstrable negligence is present [6]. Informed consent remains a critical issue, as many patients struggle to fully grasp medical risks, often citing inadequate counseling and failure to receive clear explanations before procedures [12,40]. Additionally, cultural and linguistic differences contribute to communication breakdowns, with misinterpretations and inadequate culturally tailored counseling linked to higher malpractice claims [41].

Finally, research gaps and future directions highlight the need for a broader understanding of malpractice trends. Most studies focus on high-income healthcare systems, leaving low-resource settings underrepresented despite their unique medico-legal challenges [16]. Moreover, while interventions such as simulation-based training, safety checklists, and legal reforms have been proposed, there is a lack of comprehensive research assessing their long-term impact on litigation reduction [37]. Another critical gap is the underreporting of near-miss events, which, though they do not always lead to litigation, remain undocumented, preventing a full understanding of how to best prevent malpractice claims [15].

### 4.5. Implications of Findings

The high litigation rate related to failed sterilization highlights the need for improved patient counseling and informed consent practices to mitigate medico-legal risks. The frequency of legal disputes in gynecology, particularly those arising from complications in sterilization procedures, underscores the importance of clear communication between healthcare providers and patients. Additionally, inadequate documentation and procedural deviations often lead to indefensible claims, reinforcing the necessity of standardized protocols and adherence to evidence-based practices. A multidisciplinary approach involving healthcare providers, risk management teams, and legal professionals is crucial in minimizing malpractice exposure. The impact of system-level failures, including delays in response times, lack of interdisciplinary coordination, and resource constraints, should not be underestimated in discussions on malpractice trends.

### 4.6. Limitations

This study is limited by variations in malpractice laws across different countries, which may impact the generalizability of findings. Additionally, retrospective data analysis may not capture recent trends in litigation, as many settlements occur outside of formal legal proceedings and may not be documented in publicly available legal databases. Another limitation arises from jurisdictional differences in defining and handling medical negligence, as standards of care and medico-legal responsibilities vary significantly between common law and civil law systems. Future research should explore region-specific malpractice trends and assess the impact of alternative dispute resolution mechanisms in reducing litigation rates. Furthermore, there are limited data on malpractice in low-resource settings, where different factors, such as inadequate infrastructure or lower access to advanced medical technologies, may contribute to adverse patient outcomes.

### 4.7. Future Recommendations

To mitigate malpractice risks in obstetrics and gynecology, healthcare institutions should integrate real-time risk assessment tools, enhance simulation-based training for obstetric emergencies, and adopt structured communication protocols to improve patient safety. Hospitals and medical organizations must prioritize comprehensive training programs that emphasize risk reduction strategies, procedural adherence, and ethical considerations in patient care. Implementing structured informed consent procedures that ensure patients fully understand treatment risks, alternatives, and potential complications could help prevent legal disputes. Additionally, expanding legal education for healthcare providers may reduce defensive medicine practices, allowing clinicians to balance patient-centered care with legal compliance. Future studies should evaluate the long-term effectiveness of clinical risk management strategies in reducing litigation and improving patient safety outcomes.

## 5. Conclusions

This article navigated the complex terrain of obstetric malpractice, shedding light on stakeholders’ expectations, legal implications, and clinical considerations. These findings highlight the multifaceted nature of Obstetric malpractice and underscore the importance of addressing various factors, including clinical decision-making, communication, surgical proficiency, supervision, and documentation, to enhance patient safety and mitigate the risk of litigation. From the anticipation of a perfect outcome to the legal ramifications of malpractice, each aspect of obstetric care was scrutinized. Stakeholders, including patients, healthcare providers, insurers, and legal professionals, play pivotal roles in shaping the discourse surrounding malpractice

Defining obstetric malpractice reveals a breach of duty when standards of care are not met, leading to avoidable harm. Legal landscapes were explored, emphasizing the far-reaching consequences for all parties involved. Gynecological considerations further elucidated the challenges posed by unnecessary procedures and inadequate documentation. The review concluded with a call for collaboration, education, and accountability to mitigate the impact of malpractice and uphold patient safety.

The repercussions of malpractice extend far beyond the confines of the hospital or clinic, often culminating in protracted legal battles with profound implications for all parties involved. Plaintiffs seek redress for their suffering, while defendants face threats to their professional reputation and financial stability. Insurance companies and healthcare facilities must balance cost containment with fair compensation for patients. Legal practitioners operate in a framework where precedent and evolving healthcare policies shape litigation outcomes.

Healthcare facilities must implement strong clinical risk management activities that address the organization-wide challenges in reducing medical errors. This includes process standardization, continuous monitoring of healthcare professionals’ competence, and ensuring the adequacy of medical equipment and devices. Many malpractice cases stem from systemic failures rather than isolated errors made by individual practitioners. Thus, a broader focus on hospital-wide patient safety initiatives, workforce training, and robust administrative oversight is necessary to mitigate risks.

## Figures and Tables

**Table 1:** Malpractice litigation pathways in obstetrics and gynecology.

	Key Factors Leading to Malpractice Litigation
Obstetrics	- Failure to meet care standards (e.g., during labor) - Mismanagement of complications (e.g., shoulder dystocia, fetal distress) - Deficient patient counseling - Inadequate documentation
Gynecology	- Failed sterilization procedures (19%) - Uterine perforation (8%) - Urinary tract injuries (6%) - Endoscopic surgery complications (3%) - Lack of informed consent (e.g., ovary removal) - Inadequate documentation (24%)
